# Assessment of potential human health risks in aquatic products based on the heavy metal hazard decision tree

**DOI:** 10.1186/s12859-022-04603-3

**Published:** 2022-02-17

**Authors:** Hao-Hsiang Ku, Pinpin Lin, Min-Pei Ling

**Affiliations:** 1 Institute of Food Safety and Risk Management, National Taiwan Ocean University, Keelung City, 20224 Taiwan; 2 Department of Food Science, National Taiwan Ocean University, Keelung City, 20224 Taiwan; 3 National Institute of Environmental Health Sciences, National Health Research Institutes, Zhunan Town, Miaoli County, 35053 Taiwan

**Keywords:** Decision tree, Heavy metal, Potential human health risk, Food safety risk assessment

## Abstract

**Background:**

Naturally existing and human-produced heavy metals are released into the environment and cannot be completely decomposed by microorganisms, but they continue to accumulate in water and sediments, causing organisms to be exposed to heavy metals.

**Results:**

This study designs and proposes heavy metal hazard decision trees for aquatic products, which are divided into seven categories including pelagic fishes, inshore fishes, other fishes, crustaceans, shellfish, cephalopods, and algae. Based on these classifications, representative fresh and processed seafood products are at the root of the heavy metal hazard decision trees. This study uses 2,107 cases of eating 556 cooked fresh or processed seafood product samples. The constructions of the proposed decision trees consist of 12 heavy metals, which include inorganic arsenic (iAs), cadmium (Cd), cobalt (Co), chromium (Cr), copper (Cu), iron (Fe), manganese (Mn), nickel (Ni), lead (Pb), strontium (Sr), thallium (Tl), and zinc (Zn). The heavy metal concentrations in cooked fresh and processed seafood product samples are subjected to a food safety risk assessment.

**Conclusions:**

The results indicate the relationships among the seven categories of aquatic products, the relationships among 12 heavy metals in aquatic products, and the relationships among potential human health risks. Finally, the proposed heavy metal hazard decision trees for aquatic products can be used as a reference model for researchers and engineers.

## Background

Taiwan is surrounded by the sea, with different ocean currents flowing and meeting one another, so it has abundant fish and aquatic resources. A past study showed that the people living in Taiwan consume an average of 24.62 kg of aquatic products each year, which is more than double the average annual consumption of 12.87 kg by the global population [1]. In Taiwan, people can easily buy seven categories of aquatic products at fish markets or supermarkets in counties or cities. This means that aquatic products have significant importance in the daily diet of the people in Taiwan. In recent decades, heavy metal pollutions in the environment and food have been considered a global problem [2, 3]. Naturally existing and human-produced heavy metals are released into the environment and cannot be completely decomposed by microorganisms, but they continue to accumulate in water and sediments, causing organisms to be exposed to heavy metals [4–6]. Aquatic organisms can be exposed to heavy metals in multiple ways, such as through functional groups on biofilms and heavy metal bonding, gill filtration, and ingestion. When the human body ingests aquatic products, the product passes through the food chain, and the heavy metals in the product are accumulated in the bodies of human beings [7, 8]. The excessive intake of heavy metals may cause serious health problems in people. The health problems arising from different heavy metals in the body vary according to the different types of metals, exposure time, and exposure dose [9, 10]. Therefore, it is necessary to assess the potential health risks related to heavy metals exposure in humans resulting from the intake of aquatic products.

The metal elements and inorganic arsenic (iAs) levels in fish were measured by the Core Chemical Analysis Laboratory, National Institute of Environmental Health Science, National Health Research Institutes (NIEHS, NHRI), Taiwan. For metal elements, the fish samples were digested in nitric acid with microwave and analyzed by an inductively coupled plasma-mass spectrometer. For iAs, the fish samples were extracted in nitric acid with sonication and analyzed by an inductively coupled plasma-mass spectrometer.

The estimation models are based on the Hazard Quotient (HQ) and target cancer risk of the United States Environmental Protection Agency (USEPA). These models are used to evaluate the potential health risks related to heavy metals exposure in humans through the ingestion of aquatic products [5, 11, 12]. Risk assessments consist of hazard identification, dose–response assessment, exposure assessment, and risk characterization. Hence, this study discusses the relationship between risk assessments and maximum intakes.

1. Risk assessment. To assess the risk of people’s exposure to heavy metals due to the ingestion of aquatic products in Taiwan [1]. This study integrates exposure analysis and dose–response results to compute and evaluate the Estimated Daily Intake (EDI), the Hazard Quotient (HQ), and the target cancer risk, which depend on the concentrations of heavy metals in seven categories of aquatic products. The aquatic products in this study are defined as those that people can easily buy at fish markets or supermarkets in counties or cities in Taiwan.
2. Maximum intake. After evaluating the risk assessment, this study computes the Acceptable Daily Intake (ADI) and examines the relationships among seven categories of aquatic products, the relationships among 12 heavy metals in aquatic products, and the relationships among potential human health risks [8, 13].

Regarding the aforementioned issues, this study designs and proposes heavy metal hazard decision trees for aquatic products, which are divided into seven categories, and examines the relationships among the seven categories of aquatic products, the relationships among 12 heavy metals in aquatic products, and the relationships among potential human health risks. The study indicates the risk assessments and maximum intakes of ADI.

In recent years, increasingly different aquatic products, such as large fishes, farmed fishes, algae, shellfish, crustaceans, cephalopods, and seafood products, have been investigated and researched to determine the concentrations of heavy metals in them [14–16].

Olmedo et al*.* discussed how levels of mercury (Hg), cadmium (Cd), lead (Pb), tin (Sn), and arsenic (As) have been found in fresh, canned, and frozen fishes and shellfish products and compared the levels with the maximum levels [17]. The authors surveyed a total of 485 samples of the 43 most frequently consumed fish and shellfish species in Andalusia to analyze their toxic elements content, including Hg, Cd, Pb, Sn, and As. The study recommended the provisional tolerable weekly intake (PTWI) or the benchmark dose lower confidence limit (BMDL) for a certain toxic effect. Results indicated that (1) there were high concentrations of Hg in predatory fish, (2) the Cd concentration in shellfish was higher than that in fish, (3) the Pb concentration in fishes other than frozen soles was almost negligible, (4) the Sn concentration in canned aquatic products was far below the limit standard value, and (5) fresh and frozen shrimps accumulated higher As concentrations than other species.

Qin et al*.* surveyed the concentrations of 28 trace elements, including lithium (Li), vanadium (V), chromium (Cr), manganese (Mn), iron (Fe), nickel (Ni), copper (Cu), molybdenum (Mo), zinc (Zn), selenium (Se), strontium (Sr), cobalt (Co), aluminum (Al), titanium (Ti), As, Cd, antimony (Sb), barium (Ba), Hg, Pb, uranium (U), silver (Ag), beryllium (Be), thallium (Tl), gallium (Ga), tellurium (Te), Sn, and thulium (Tm), in three farmed cyprinid fish species from Northeast China [18]. Because the detection rates of Co, Be, Ga, Ag, Sn, Te, Tm, and Tl were less than 50%, no subsequent comparison was made. The concentrations of trace elements in the three farmed fishes from high to low were Zn (7.9 mg/kg) > Fe (6.71) > Al (6.30) > Sr (1.171) > Cu (0.293) > Se (0.243) > Ti (0.237) > Ba (0.193) > Pb (0.172) > Mn (0.136) > Cr (0.121) > Ni (0.119) > As (0.096) > V (0.019) > Mo (0.015) > Hg (0.013) > Li (0.01) ≈ Cd (0.01) ≈ Sb (0.01) > U (0.004). The results were below the limit standard set by the Chinese government: As was 0.5 mg/kg, Hg was 0.3 mg/kg, Pb was 0.5 mg/kg, Cd was 0.1 mg/kg, Cr was 2.0 mg/kg, and Cu was 50 mg/kg. Hence, results indicated that (1) the fish samples with 0.56% As, 1.13% Cd, and 9.04% Pb were above the maximum residue limit (MRL), (2) the hazard index and target cancer risk suggested that farmed fishes were generally safe for consumers, and (3) As, Cd, and Pb should be the most important monitored targets. The study concluded that it was safe for consumers to eat farmed fishes from Northeast China.

Santo et al*.* surveyed an analysis of Cd and Pb contamination in fishes (Centropomus undecimalis and Mugil brasiliensis), mussels (Mytella guyanensis) and shrimps (Penaeus brasiliensis) in the municipality of São Francisco do Conde, located in Todos os Santos Bay, Brazil [19]. Among the 47 samples, 11 samples had at least one metal exceeding the Cd and Pb limit standards in aquatic products established by Brazil (Cd: 1 μg/g; Pb: 2 μg/ g). However, all fishes were within the limit standard. Hence, the study speculated that shellfish were more likely to accumulate heavy metals in the body than fishes and shrimps.

Copat et al*.* evaluated the concentrations of As, Cd, Cr, Pb, Mn, Ni, V, and Zn in fishes and shellfish from the Gulf of Catania. The results showed that the concentrations of As, Cd, Cr, Pb, Mn, Ni, V, and Zn were 3.135–11.024 mg/kg, 0.0004–0.0013 mg/kg, 0.007–0.015 mg/kg, 0.003–0.021 mg/kg, 0.122–2.454 mg/kg, 0.011–0.086 mg/kg, 0.074–0.149 mg/kg, and 3.418–6.580 mg/kg in fishes and 1.528 mg/kg, 0.0053 mg/kg, 0.245 mg/kg, 0.071 mg/kg, 4.255 mg/kg, 0.327 mg/kg, 0.497 mg/kg, and 7.625 mg/kg in shellfish, respectively [7]. The study combined the concentrations of heavy metals in aquatic products with the food intake of Italian adults and children to estimate the EDI of heavy metals in aquatic products and then compared the results with the human tolerable dose. The HQ value of heavy metals in all species was less than 1. Results indicated that (1) the consumption rates recommended to minimize risks to human health had been estimated, (2) the target hazard quotient values suggested that humans should minimize meals/week according to the species analyzed, (3) consuming fish with inorganic arsenic (iAs) concentrations was assumed to have an acceptable risk for cancer, and (4) the fishes and shellfish from the Gulf of Catania were safe to eat.

The assessment of potential human health risks is an important issue in the world. The traditional assessment depends on surveying the food chemicals of multiple foods based on a total diet study (TDS). TDS estimates the dietary exposures to food chemicals or nutrients and assesses health risks. It requires significant resources and funds to conduct detailed surveys and rigorous inspections. Hence, it is important to increase the value of assessments by constructing multiple decision trees to replace many kinds of inspection reports. This study designs and proposes heavy metal hazard decision trees for aquatic products, which are divided into seven categories including pelagic fishes, inshore fishes, other fishes, crustaceans, shellfish, cephalopods, and algae. This study collects 556 cooked fresh or processed seafood product samples. The constructions of the proposed heavy metal hazard decision trees include 12 heavy metals.

## Results

This study constructs heavy metal hazard decision trees, including seven sub-heavy metal hazard decision trees, to estimate and describe the relationship between the seven categories of aquatic products and the long-term incorporation of iAs, Cd, Co, Fe, Sr, Tl, and Zn heavy metals. The seven categories of aquatic products are pelagic fishes, inshore fishes, other fishes, crustaceans, shellfish, cephalopods, and algae. Aquatic products in this study are defined as those that people can easily buy at fish markets or supermarkets in counties or cities in Taiwan. The subjects are adults aged 19–64 years old [20–22]. The input dataset is total exposure, which is the category of aquatic products consumed from the databases of the Fishery Statistics Annual Report in Taiwan and the National Food Consumption Database in Taiwan [23, 24]. This study includes 2,529 cases of food intake. The heavy metal hazard decision tree has a two-stage construction. In the first stage, the heavy metal decision tree model is constructed by selecting and adjusting the factors based on 422 cases. In the second stage, the model evaluates cases and constructs rules. The 2,107 cases of eating 556 cooked fresh seafood or processed seafood product samples are used to determine rules. Table 1 presents the heavy metals of samples.

**Table 1:** The heavy metals of samples (mean ± standard deviation)(mg/kg)

Classification	Pelagic fishes	Inshore fishes	Other fishes	Crustaceans	Shellfish	Cephalopods	Algae
**Number of samples**	26	45	98	32	43	16	18
iAs	0.0006 ± 0.0011	0.0023 ± 0.0036	0.0024 ± 0.0036	0.0027 ± 0.0046	0.025 ± 0.0054	0.0042 ± 0.0096	0.036 ± 0.054
Cd	0.025 ± 0.053	0.0066 ± 0.011	0.0088 ± 0.03	0.10 ± 0.16	1.25 ± 3.06	0.43 ± 0.78	0.097 ± 0.066
Co	0.003 ± 0.002	0.006 ± 0.009	0.0051 ± 0.0049	0.019 ± 0.024	0.19 ± 0.31	0.0067 ± 0.0081	0.024 ± 0.024
Cr	0.10 ± 0.16	0.14 ± 0.62	0.063 ± 0.11	0.22 ± 0.52	0.16 ± 0.23	0.094 ± 0.10	0.20 ± 0.18
Cu	1.23 ± 1.61	1.31 ± 3.31	2.97 ± 8.01	9.34 ± 4.24	6.98 ± 8.80	5.34 ± 4.36	2.61 ± 3.44
Fe	5.15 ± 4.06	5.68 ± 7.97	6.17 ± 8.90	10.45 ± 14.42	71.46 ± 67.25	2.36 ± 2.34	39.84 ± 52.52
In	0.0022 ± 0.0073	0.0026 ± 0.0094	0.0063 ± 0.024	0.0017 ± 0.0037	0.0025 ± 0.0061	0.0037 ± 0.0066	0.012 ± 0.027
Mn	0.09 ± 0.03	0.16 ± 0.22	0.35 ± 1.23	0.83 ± 0.86	3.57 ± 2.65	0.20 ± 0.14	6.19 ± 9.68
Ni	0.05 ± 0.07	0.079 ± 0.29	0.037 ± 0.072	0.15 ± 0.27	0.55 ± 0.56	0.055 ± 0.066	0.12 ± 0.091
Pb	0.002 ± 0.002	0.0052 ± 0.0056	0.0079 ± 0.014	0.013 ± 0.019	0.082 ± 0.071	0.015 ± 0.017	0.13 ± 0.11
Sr	0.27 ± 0.16	0.80 ± 1.04	2.47 ± 9.12	14.12 ± 23.33	6.29 ± 3.59	2.63 ± 1.18	20.90 ± 21.47
Tl	0.0002 ± 0.0002	0.00037 ± 0.00031	0.00069 ± 0.001	0.00031 ± 0.00026	0.00074 ± 0.00073	0.00020 ± 0.00012	0.00057 ± 0.00050
Zn	4.78 ± 1.45	4.86 ± 2.91	6.49 ± 7.23	23.87 ± 13.85	36.50 ± 34.14	12.51 ± 3.85	3.88 ± 3.43

The principle behind the classification of aquatic products is to develop a representative list of fresh aquatic products roughly divided into seven categories based on Taiwan Food and Drug Administration (TFDA) announcements. The categories are (1) pelagic fishes, including shark, sailfish, tuna, and oil fish;

(2) inshore fishes, including cod, bonito, sea bream, catfish, anglerfish, flounder, mullet, stingray, hairtail, anchovy, scorpionfish, mullet, catfish, sturgeon, goldfish, eel, and barracuda; (3) other fishes, which are fishes other than pelagic fishes and inshore fishes; (4) cephalopods; (5) crustaceans; (6) algae; and (7) shellfish.

### Inorganic arsenic (iAs)

**Fig. 1:**
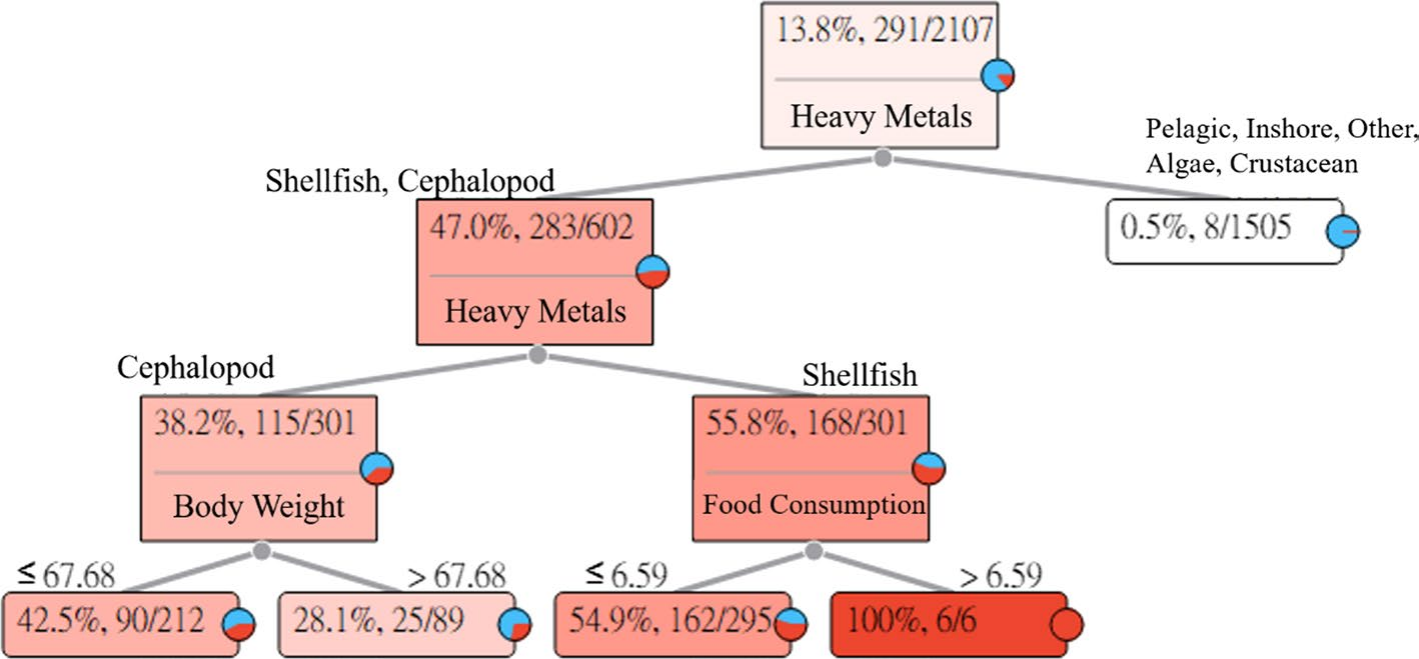
Inorganic Arsenic (iAs) Heavy Metal Hazard Decision Tree

The long-term intake of aquatic products with iAs may cause hyperpigmentation and keratosis follicularis in humans. The iAs Heavy Metal Hazard Decision Tree explains and describes the relationships between the seven categories of aquatic products and symptoms. The results of the estimation are shown in Fig. 1.

Figure 1 indicates that the intake of iAs in pelagic fishes, inshore fishes, other fishes, algae, and crustaceans will not easily cause hyperpigmentation and keratosis follicularis in adults. However, the intake of iAs in shellfish and cephalopods has the probability of causing hyperpigmentation and keratosis follicularis in adults.

1. The intake of cephalopods. Among the 212 subjects whose body weights were less than or equal to 67.68 (kg), 90 subjects had HQ values greater than 1. For an adult whose body weight was less than or equal to 67.68 (kg) who ingested cephalopods with iAs, the risk of causing hyperpigmentation and keratosis follicularis was 42.5%. In addition, among the 89 subjects whose body weights were greater than 67.68 (kg), 25 subjects had HQ values greater than 1. For an adult whose body weight was greater than 67.68 (kg) who ingested cephalopods with iAs, the risk of causing hyperpigmentation and keratosis follicularis was 28.1%.
2. The intake of shellfish. Among the 295 subjects whose food intakes were less than or equal to 6.59 (g/week), 162 subjects had HQ values greater than 1. For an adult whose intake of shellfish with iAs was less than or equal to 6.59 (g/week), the risk of causing hyperpigmentation and keratosis follicularis was 54.9%. In addition, among the six subjects whose food intakes were greater than 6.59 (g/week), six subjects had HQ values greater than 1. For an adult whose intake of shellfish with iAs was greater than 6.59 (g/week), the risk of causing hyperpigmentation and keratosis follicularis was 100%.

### Cadmium (Cd)

**Fig. 2:**
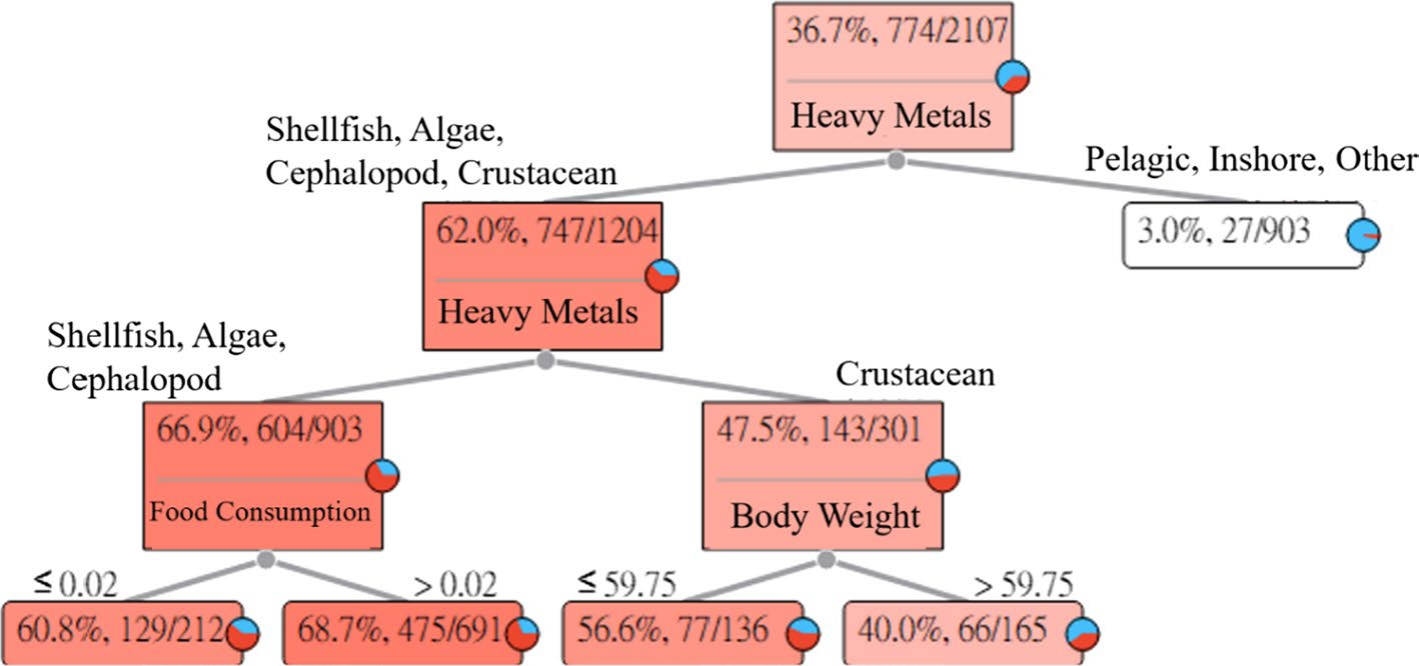
Cadmium (Cd) Heavy Metal Hazard Decision Tree

The long-term intake of aquatic products with Cd may cause kidney diseases in humans. The Cd Heavy Metal Hazard Decision Tree explains and describes the relationships between the seven categories of aquatic products and symptoms. The results of the estimation are shown in Fig. 2.

Figure 2 indicates that the intake of Cd in pelagic fishes, inshore fishes, and other fishes will not easily cause kidney diseases in adults. However, the intake of Cd in shellfish, algae, cephalopods, and crustaceans has the probability of causing kidney diseases in adults.

(1) The intake of shellfish, algae, and cephalopods. Among the 212 subjects whose food intakes were less than or equal to 0.02 (g/week), 129 subjects had HQ values greater than 1. For an adult whose intake of shellfish, algae, and cephalopods with Cd was less than or equal to 0.02 (g/week), the risk of causing kidney diseases was 60.8%. In addition, among the 691 subjects whose food intakes were greater than 0.02 (g/week), 475 subjects had HQ values greater than 1. For an adult whose intake of shellfish, algae, and cephalopods with Cd was greater than 0.02 (g/week), the risk of causing kidney diseases was 68.7%.

(2) The intake of crustaceans. Among the 136 subjects whose body weights were less than or equal to 59.75 (kg), 77 subjects had HQ values greater than 1. For an adult whose body weight was less than or equal to 59.75 (kg) who ingested crustaceans with Cd, the risk of causing kidney diseases was 56.6%. In addition, among the 165 subjects whose body weights were greater than 59.75 (kg), 66 subjects had HQ values greater than 1. For an adult whose body weight was greater than 59.75 (kg) who ingested crustaceans with Cd, the risk of causing kidney diseases was 40.0%.

### Cobalt (Co)

**Fig. 3:**
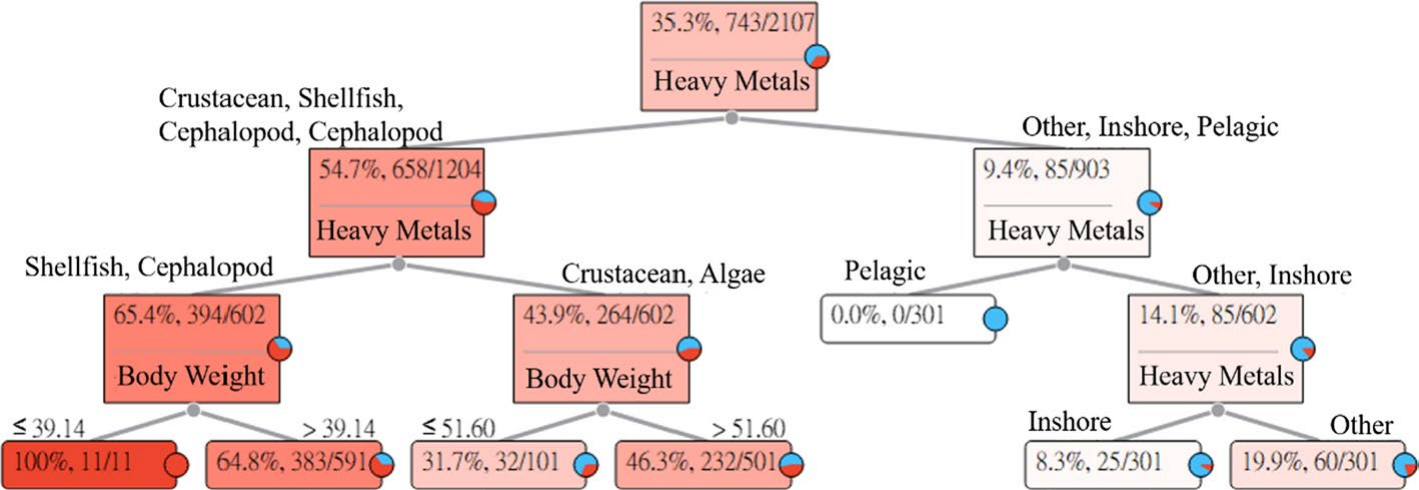
Cobalt (Co) Heavy Metal Hazard Decision Tree

The long-term intake of aquatic products with Co may cause goiter. The Co Heavy Metal Hazard Decision Tree explains and describes the relationships between the seven categories of aquatic products and symptoms. The results of the estimation are shown in Fig. 3.

Figure 3 indicates that the intake of Co in pelagic fishes, inshore fishes, and other fishes will not cause goiter in adults. However, the intake of Co in crustaceans, algae, shellfish, and cephalopods has the probability of causing goiter in adults.

1. The intake of shellfish and cephalopods. Among the 11 subjects whose body weights were less than or equal to 39.14 (kg), 11 subjects had HQ values greater than 1. For an adult whose body weight was less than or equal to 39.14 (kg) who ingested shellfish and cephalopods with Co, the risk of causing goiter was 100%. In addition, among the 591 subjects whose body weights were greater than 39.14 (kg), 383 subjects had HQ values greater than 1. For an adult whose body weight was greater than 39.14 (kg) who ingested shellfish and cephalopods with Co, the risk of causing goiter was 64.8%.
2. The intake of crustaceans and algae. Among the 101 subjects whose body weights were less than or equal to 51.6 (kg), 32 subjects had HQ values greater than 1. For an adult whose body weight was less than or equal to 51.6 (kg) who ingested crustaceans and algae with Co, the risk of causing goiter was 31.7%. In addition, among the 501 subjects whose body weights were greater than 51.6 (kg), 232 subjects had HQ values greater than 1. For an adult whose body weight was greater than 51.6 (kg) who ingested crustaceans and algae with Co, the risk of causing goiter was 46.3%.
3. The intake of pelagic fishes. None of the 301 subjects had HQ values greater than 1. For an adult who ingested pelagic fishes with Co, the risk of causing goiter was 0%.
4. The intake of inshore fishes. Among the 301 subjects, 25 subjects had HQ values greater than 1. For an adult who ingested inshore fishes with Co, the risk of causing goiter was 8.3%.
5. The intake of other fishes. Among the 301 subjects, 60 subjects had HQ values greater than 1. For an adult who ingested other fishes with Co, the risk of causing goiter was 19.9%.

### Iron (Fe)

**Fig. 4:**
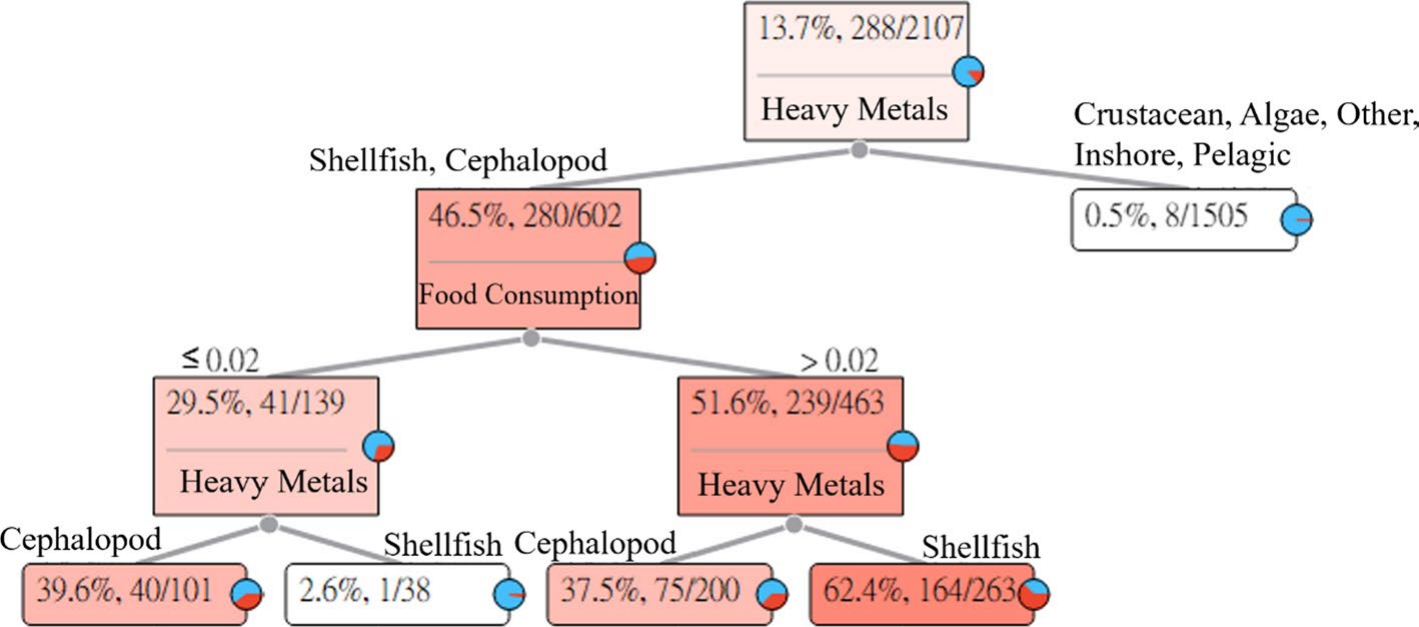
Iron (Fe) Heavy Metal Hazard Decision Tree

The long-term intake of Fe in aquatic products may cause gastrointestinal disorders. The Fe Heavy Metal Hazard Decision Tree explains and describes the relationships between the seven categories of aquatic products and symptoms. The results of the estimation are shown in Fig. 4.

Figure 4 indicates that the intake of Fe in crustaceans, algae, other fishes, inshore fishes, and pelagic fishes will not easily cause gastrointestinal disorders in adults. However, the intake of Fe in shellfish and cephalopods has the probability of causing gastrointestinal disorders in adults.

1. The intake of shellfish and cephalopods with Fe that was less than or equal to 0.02 (g/week). Among the 101 subjects whose intake of cephalopods with Fe was less than or equal to 0.02 (g/week), 40 subjects had HQ values greater than 1. For an adult who ingested cephalopods with Fe, the risk of causing gastrointestinal disorders was 39.6%. In addition, among the 38 subjects whose intake of shellfish with Fe was less than or equal to 0.02 (g/week), one subject had HQ values greater than 1. For an adult who ingested shellfish with Fe, the risk of causing gastrointestinal disorders was 2.6%.
2. The intake of shellfish and cephalopods with Fe that was greater than 0.02 (g/week). Among the 200 subjects whose intake of cephalopods with Fe was greater than 0.02 (g/week), 75 subjects had HQ values greater than 1. For an adult who ingested cephalopods with Fe, the risk of causing gastrointestinal disorders was 37.5%. In addition, among the 263 subjects whose intake of shellfish with Fe was greater than 0.02 (g/week), 164 subjects had HQ values greater than 1. For an adult who ingested shellfish with Fe, the risk of causing gastrointestinal disorders was 62.4%.

### Strontium (Sr)

**Fig. 5:**
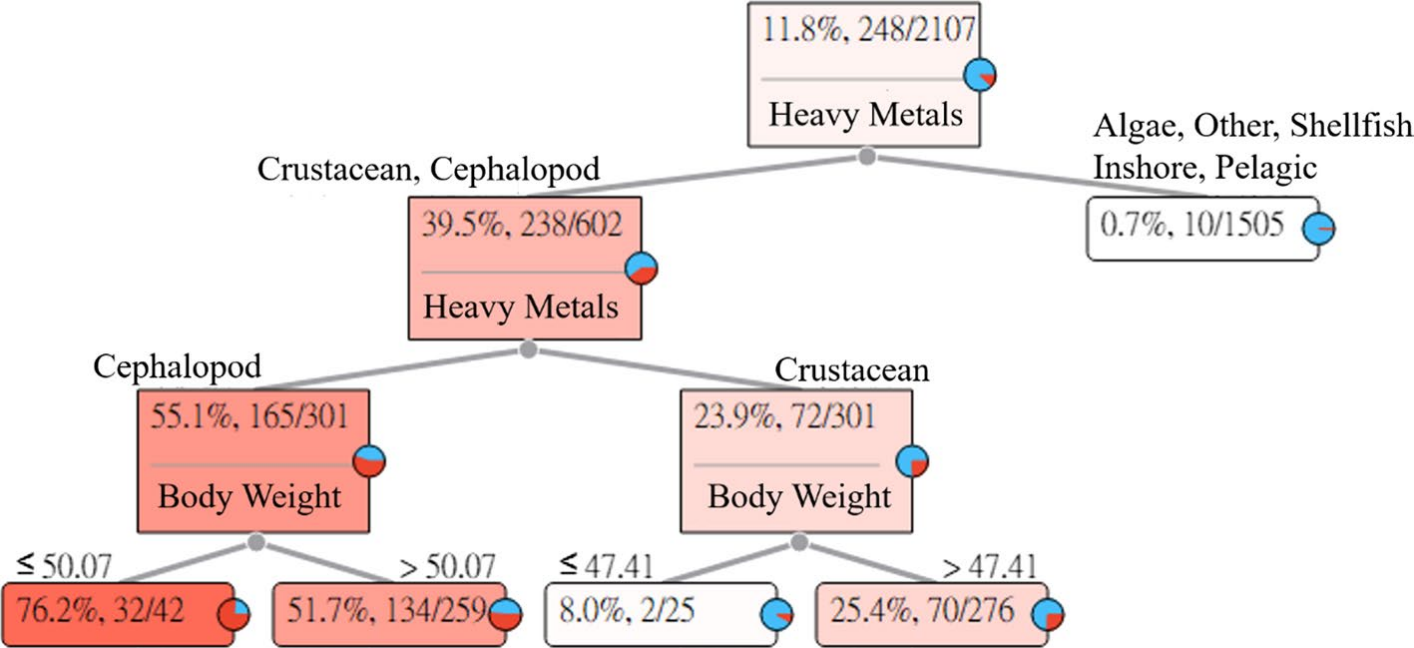
Strontium (Sr) Heavy Metal Hazard Decision Tree

The long-term intake of Sr in aquatic products may cause adult rickets. The Sr Heavy Metal Hazard Decision Tree explains and describes the relationships between the seven categories of aquatic products and symptoms. The results of the estimation are shown in Fig. 5.

Figure 5 indicates that the intake of Sr in algae, other fishes, shellfish, inshore fishes, and pelagic fishes will not easily cause rickets in adults. However, the intake of Sr in crustaceans and cephalopods has the probability of causing rickets in adults.

1. The intake of cephalopods. Among the 42 subjects whose body weights were less than or equal to 50.07 (kg), 32 subjects had HQ values greater than 1. For an adult whose body weight was less than or equal to 50.07 (kg) who ingested cephalopods with Sr, the risk of causing adult rickets was 76.2%. In addition, among the 259 subjects whose body weights were greater than 50.07 (kg), 134 subjects had HQ values greater than 1. For an adult whose body weight was greater than 50.07 (kg) who ingested cephalopods with Sr, the risk of causing adult rickets was 51.7%.
2. The intake of crustaceans. Among the 25 subjects whose body weights were less than or equal to 47.41 (kg), two subjects had HQ values greater than 1. For an adult whose body weight was less than or equal to 47.41 (kg) who ingested crustaceans with Sr, the risk of causing adult rickets was 8.0%. In addition, among the 276 subjects whose body weights were greater than 47.41 (kg), 70 subjects had HQ values greater than 1. For an adult whose body weight was greater than 47.41 (kg) who ingested crustaceans with Sr, the risk of causing adult rickets was 25.4%.

### Thallium (Tl)

**Fig. 6:**
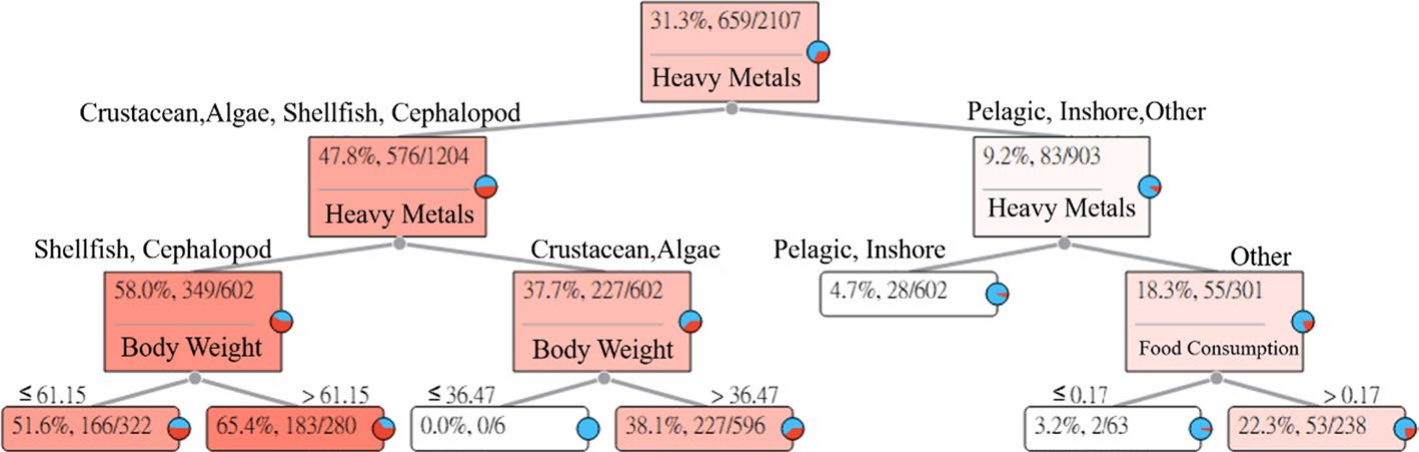
Thallium (Tl) Heavy Metal Hazard Decision Tree

The long-term intake of Tl in aquatic products may cause perifollicular atrophy. The Tl Heavy Metal Hazard Decision Tree explains and describes the relationships between the seven categories of aquatic products and symptoms. The results of the estimation are shown in Fig. 6.

Figure 6 indicates that the probability of the intake of Tl in pelagic fishes, inshore fishes, and other fishes causing perifollicular atrophy in adults is about 9.2%, while the probability of the intake of Tl in crustaceans, shellfish, cephalopods, and algae causing perifollicular atrophy in adults is about 47.8%.

1. The intake of shellfish and cephalopods. Among the 322 subjects whose body weights were less than or equal to 61.15 (kg), 166 subjects had HQ values greater than 1. For an adult whose body weight was less than or equal to 61.15 (kg) who ingested shellfish and cephalopods with Tl, the risk of causing perifollicular atrophy was 51.6%. In addition, among the 280 subjects whose body weights were greater than 61.15 (kg), 183 subjects had HQ values greater than 1. For an adult whose body weight was greater than 61.15 (kg) who ingested shellfish and cephalopods with Tl, the risk of causing perifollicular atrophy was 65.4%.
2. The intake of crustaceans and algae. None of the six subjects whose body weights were less than or equal to 36.47 (kg) had HQ values greater than 1. For an adult whose body weight was less than or equal to 36.47 (kg) who ingested crustaceans and algae with Tl, the risk of causing perifollicular atrophy was 0%. In addition, among the 596 subjects whose body weights were greater than 36.47 (kg), 227 subjects had HQ values greater than 1. For an adult whose body weight was greater than 36.47 (kg) who ingested crustaceans and algae with Tl, the risk of causing perifollicular atrophy was 38.1%.
3. The intake of pelagic fishes and inshore fishes. Among the 602 people, 28 subjects had HQ values greater than 1. For an adult who ingested pelagic fishes and inshore fishes with Tl, the risk of causing perifollicular atrophy was 4.7%.
4. The intake of other fishes. Among the 63 subjects whose food intakes were less than or equal to 0.17 (g/week), two subjects had HQ values greater than 1. For an adult whose intake of other fishes with Tl was less than or equal to 0.17 (g/week), the risk of causing perifollicular atrophy was 3.2%. In addition, among the 238 subjects whose food intakes were greater than 0.17 (g/week), 53 subjects had HQ values greater than 1. For an adult whose intake of other fishes with Tl was greater than 0.17 (g/week), the risk of causing perifollicular atrophy was 22.3%.

### Zinc (Zn)

**Fig. 7:**
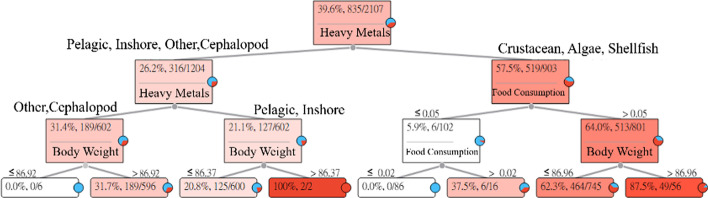
Zinc (Zn) Heavy Metal Hazard Decision Tree

The long-term intake of Zn in aquatic products may reduce superoxide dismutase activity in red blood cells. The Zn Heavy Metal Hazard Decision Tree explains and describes the relationships between the seven categories of aquatic products and symptoms. The results of the estimation are shown in Fig. 7.

Figure 7 indicates that the probability of the intake of Zn in pelagic fishes, inshore fishes, other fishes, and cephalopod reducing superoxide dismutase activity in red blood cells in adults is about 26.2%, while the probability of the intake of Zn in crustaceans, shellfish, and algae reducing superoxide dismutase activity in red blood cells in adults is about 57.5%.

(1) The intake of other fishes and cephalopods. None of the six subjects whose body weights were less than or equal to 86.92 (kg) had HQ values greater than 1. For an adult whose body weight was less than or equal to 86.92 (kg) who ingested other fishes and cephalopods with Zn, the risk of reducing superoxide dismutase activity in red blood cells was 0%. In addition, among the 596 subjects whose body weights were greater than 86.92 (kg), 189 subjects had HQ values greater than 1. For an adult whose body weight was greater than 86.92 (kg) who ingested other fishes and cephalopods with Zn, the risk of reducing superoxide dismutase activity in red blood cells was 31.7%.

(2) The intake of pelagic fishes and inshore fishes. Among the 600 subjects whose body weights were less than or equal to 86.37 (kg), 125 subjects had HQ values greater than 1. For an adult whose body weight was less than or equal to 86.37 (kg) who ingested pelagic fishes and inshore fishes with Zn, the risk of reducing superoxide dismutase activity in red blood cells was 20.8%. In addition, among the two subjects whose body weights were greater than 86.37 (kg), both had HQ values greater than 1. For an adult whose body weight was greater than 86.37 (kg) who ingested pelagic fishes and inshore fishes with Zn, the risk of reducing superoxide dismutase activity in red blood cells was 100%.
(3) The intake of crustaceans, algae, and shellfish. None of the 86 subjects whose food intakes were less than or equal to 0.02 (g/week) had HQ values greater than 1. For an adult whose intake of crustaceans, algae, and shellfish with Zn was less than or equal to 0.02 (g/week), the risk of reducing superoxide dismutase activity in red blood cells was 0%. In addition, among the 16 subjects whose food intakes were greater than 0.02 (g/week), six subjects had HQ values greater than 1. For an adult whose intake of crustaceans, algae, and shellfish with Zn was greater than 0.02 (g/week), the risk of reducing superoxide dismutase activity in red blood cells was 37.5%.
(4) The intake of crustaceans, algae, and shellfish. Among the 745 subjects whose body weights were less than or equal to 86.96 (kg), 464 subjects had HQ values greater than 1. For an adult whose body weight was less than or equal to 86.96 (kg) who ingested crustaceans, algae, and shellfish with Zn, the risk of reducing superoxide dismutase activity in red blood cells was 62.3%. In addition, among the 56 subjects whose body weights were greater than 86.96 (kg), 49 subjects had HQ values greater than 1. For an adult whose body weight was greater than 86.96 (kg) who ingested crustaceans, algae, and shellfish with Zn, the risk of reducing superoxide dismutase activity in red blood cells was 87.5%.

## Discussion

Risk assessment includes hazard identification, dose–response assessment, exposure assessment, and risk characterization. After the Cr, Cu, Mn, Ni, and Pb values are calculated using the HQ, it is found that they will not cause adverse effects on the human body. Hence, this study focuses on explaining and constructing the heavy metal hazard decision trees of iAs, Cd, Co, Fe, Sr, Tl, and Zn. The proposed heavy metal hazard decision trees illustrate multiple suggestions and related hazards concerning the intake of aquatic products with iAs, Cd, Co, Fe, Sr, Tl, and Zn in Taiwan. The analyzed highest risks are described as follows.

1. For an adult whose intake of shellfish with iAs is greater than 6.59 (g/week), the risk of causing hyperpigmentation and keratosis follicularis is 100%.
2. For an adult whose intake of shellfish, algae, and cephalopod with Cd is greater than 0.02 (g/week), the risk of causing kidney disease is 68.7%.
3. For an adult whose body weight is less than or equal to 39.14 kg who ingests shellfish and cephalopod with Co, the risk of causing goiter is 100%.
4. For an adult whose intake of shellfish with Fe is greater than 0.02 (g/week), the risk of causing gastrointestinal disorders is 62.4%.
5. For an adult whose body weight is less than or equal to 50.07 kg who ingests cephalopods with Sr, the risk of causing adult rickets is 76.2%.
6. For an adult whose body weight is greater than 61.15 kg who ingests shellfish and cephalopods with Tl, the risk of causing perifollicular atrophy is 65.4%.
7. For an adult whose body weight is greater than 86.37 kg who ingests pelagic fishes and inshore fishes with Zn, the risk of reducing superoxide dismutase activity in red blood cells is 100%.

Therefore, it is recommended that people should eat aquatic products appropriately, pay attention to their heavy metal content, and avoid the excessive intake of heavy metals.

## Conclusions

The health problems resulting from different heavy metals in the body vary according to the different types of metals, exposure time, and exposure dose. This study designs and proposes heavy metal hazard decision trees for aquatic products, which are divided into seven categories including pelagic fishes, inshore fishes, other fishes, crustaceans, shellfish, cephalopods, and algae. Based on these classifications, representative fresh and processed seafood products are at the root of the heavy metal hazard decision trees. The study discusses 12 kinds of heavy metals, which are iAs, Cd, Co, Cr, Cu, Fe, Mn, Ni, Pb, Sr, Tl, and Zn. After the Cr, Cu, Mn, Ni, and Pb values are calculated using the HQ, it is found that they will not cause adverse effects on the human body. Hence, this study focuses on explaining and constructing the heavy metal hazard decision trees of iAs, Cd, Co, Fe, Sr, Tl, and Zn. Finally, five research recommendations are proposed for researchers.

1. To design and develop a deep learning mechanism for the risk assessment of aquatic products. It can make the assessment results more accurate and predictable.
2. To study the interactive influence model of diversified diets. Different diet patterns could have different risk assessments.
3. To study the relationships between individual and group risk assessments. This analysis could identify high-risk groups and their eating habits.
4. To study the relationships among environmental pollutions, heavy metal poisons, and fishing conditions based on the fluctuations and differences in the contained heavy metals. The changes and trends can be used to obtain more accurate estimates of the dietary risks and safety of aquatic products.
5. To survey the conditions of different aquatic products within different sea areas in Taiwan. This analysis could help consumers to know the food safety of aquatic products in a specific sea area.

## Methods

**Fig. 8:**
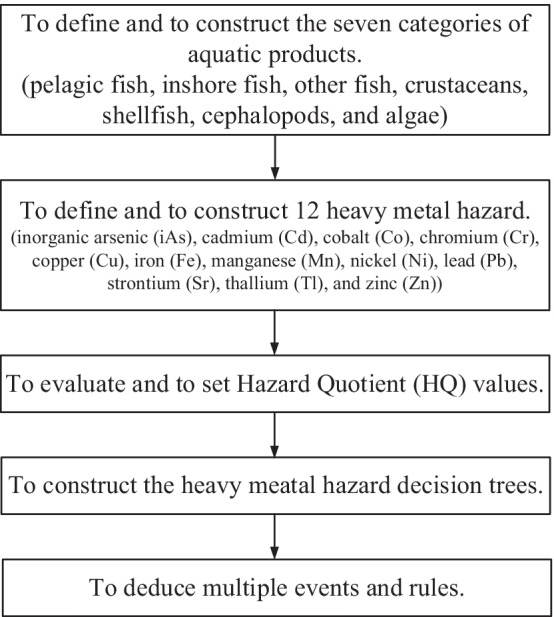
The processes of constructing the heavy metal hazard decision trees

A decision tree is a kind of a predictive model for classification and prediction in machine learning. It represents a mapping relationship between the object attributes and the object values [25–27]. Each node in the tree represents an object, and each path represents a possible attribute value. Each leaf node corresponds to the value of the object represented by the path from the root to the leaf node. Decision tree is a frequently used technique in data mining, which can be used to analyze data and make predictions [28–31]. The processes of constructing the heavy metal hazard decision trees are illustrated in Fig. 8.

The input dataset is total exposure, which is the category of aquatic products consumed from the databases of the Fishery Statistics Annual Report in Taiwan and the National Food Consumption Database in Taiwan, combined with the aquatic product sampling method. After sampling, homogenization, and cooking, the concentrations of heavy metals in various aquatic products are measured. Formula (1) calculates the food intake to estimate the EDI of heavy metals in the aquatic products ingested by each exposed group [5].

1 $${\text{EDI}}_{\text{ij}}{ = }\sum_{\text{j} = {1}}^{\text{n}} \, \frac{{\text{C}}_{\text{i}} \times {\text{CR}}_{\text{ij}} \times {10}^{-3}}{{\text{BW}}_{\text{j}}}$$

*EDI*_*ij*_ is the average daily estimated intake of heavy metals in the aquatic product, *i*, ingested by each exposed group, *j* (mg/kg bw/day). *C*_*i*_ is the concentration of heavy metals in the cooked aquatic product, *I* (mg/kg). *CR*_*ij*_ is the food intake of the aquatic product, *i*, ingested by each exposed group, *j* (consumption rate, g/day). *BW*_*j*_ is the body weight of each exposed group, *j* (kg).

Based on these classifications, representative fresh and processed seafood products are at the root of the heavy metal hazard decision trees. For the specific exposure, the Hazard Quotient (HQ) is obtained by comparing the EDI and the reference dose of the hazardous substance, which also represents the ratio of the toxic substance dose to the tolerable dose ingested by the consumer through the same route for a long time. When the HQ value is less than or equal to 1, it means that the health risk caused by the intake of a heavy metal is within an acceptable range. If the HQ value is greater than 1, it means that the intake of a heavy metal is in excess and may have adverse effects on the consumer’s health. There is a risk of poisoning or illness [11, 12]. The formula for the HQ is denoted as Formula (2).

2 $${HQ}_{ij}=\frac{{EDI}_{ij}}{HBGV}$$

*HQ*_*ij*_ is the HQ of heavy metals in the aquatic products, *i*, ingested by each exposed group, *j*. *EDI*_*ij*_ is the estimated average daily intake of heavy metals in the aquatic product, *i*, of each exposure group, *j* (mg/kg bw/day). HBGV is the abbreviation of Health-Based Guidance Value. Hence, HQ can be denoted as Formula (3):

3 $${HQ}_{ij}=\frac{{C}_{i}\times {CR}_{ij}}{{BW}_{j}\times {RfD}_{i}}$$

*C*_*i*_ is the concentration of heavy metals in the cooked aquatic product, *i* (mg/kg). *CR*_*ij*_ is the intake of aquatic product, *i*, in each exposed group, *j* (consumption rate, g/day). *BW*_*j*_ is the body weight of each exposed group, *j* (kg). *RfD* is the abbreviation of Reference Dose, which is mg/kg bw/day.

This study designs and proposes heavy metal hazard decision trees for aquatic products, which are divided into seven categories including pelagic fishes, inshore fishes, other fishes, crustaceans, shellfish, cephalopods, and algae. The heavy metal hazard decision trees are based on the HQ to assess the potential health risks of human exposure to heavy metals through the ingestion of aquatic products. The subjects are adults aged 19–64 years old [20–22]. The heavy metals examined are iAs, Cd, Co, Cr, Cu, Fe, Mn, Ni, Pb, Sr, Tl, and Zn. After the Cr, Cu, Mn, Ni, and Pb values are calculated using the HQ, it is found that they will not cause adverse effects on the human body. Hence, this study explains and constructs the heavy metal hazard decision trees of iAs, Cd, Co, Fe, Sr, Tl, and Zn. Table 2 illustrates the algorithm of the proposed heavy metal hazard decision trees.

**Table 2:** The algorithm of the heavy metal hazard decision tree

Heavy metal hazard decision tree	
**Begin** Instances aquaticProducts = [‘pelagic fish’, ‘inshore fish’, ‘other fishes’, ‘crustaceans’, ‘shellfish’, ‘cephalopods’, ‘algae’] heavyMetals = [‘inorganic arsenic (iAs)’, ‘cadmium (Cd)’, ‘cobalt (Co)’, ‘chromium (Cr)’, ‘copper (Cu)’, ‘iron (Fe)’, ‘manganese (Mn)’, ‘nickel (Ni)’, ‘lead (Pb)’, ‘strontium (Sr)’, ‘thallium (Tl)’, ‘zinc (Zn)’] attributes = [‘heavy_metal’, ‘food_intake’, ‘bw’, ‘rfd’, ‘HQ’, ‘EDI’, ‘ADI’] symptom = [‘hyperpigmentation and keratosis follicularis’, ‘kidney diseases’, ‘goiter’, ‘lung cancer’, ‘Alzheimer’s disease and cardiac disease’, ‘gastrointestinal disorders’, ‘parkinsonism’, ‘chronic bronchitis’, ‘intellectual disability’, ‘adult rickets’, ‘perifollicular atrophy’, ‘reduce superoxide dismutase activity in red blood cells’] //depending on different heavy metals Input: Dataset with attribute values Create empty “Root” node in the HMHDT model. //Heavy Metal Hazard Decision Tree (HMHDT) repeat (decisionTreeClassifier(criterion = "**Entropy**")) evaluateInformationGain(Gain(S,A), SplitInfoA(S), GainRatio(A)) classAggregated = aggregateMultipleFeatures(Instance.aquaticProducts, Instance.heavyMetals, Instance.attributes) bestAttributes = FindBestSplitAttributeAtEachLevel(ClassAggregated) calculateRiskRatio( Instance.attributes.[HQ, EDI, ADI]) updateHMHDT(HMHDT, bestAttribute) HMHDT = IdentifyDataForNextLevel(HMHDT, bestAttributes) until no node left to expand OR depth of tree in HMHDT has reached maxDepth mapping(Instance.heavyMetals, Instance.symptom) Output HMHDT **End**	

Information Gain, Gain(S, A), is the response for selecting an attribute to be a node of the decision tree. When the Gain(S, A) value is larger, the messiness of the data is smaller. This attribute is more suitable as a node. Split Information, SplitInfo_A_(S), is the entropy of the distribution of instances into branches. Gain Ratio, GainRatio(A), reduces its bias attributes. It takes the number and size of branches into account when choosing, which corrects the value by the Information Gain and the Split Information. It makes the decision tree model more accurate. Formulas (4), (5), and (6) illustrate the construction of the heavy metal hazard decision trees.

4 $$Gain\left(S,A\right)=Entropy\left(S\right)-\sum_{j=1}^{v}\frac{\left|{S}_{j}\right|}{\left|S\right|}Entropy({S}_{j})$$

5 $${SplitInfo}_{A}\left(S\right)=-\sum_{j=1}^{v}\frac{\left|{S}_{j}\right|}{\left|S\right|}\times {\mathrm{log}}_{2}(\frac{{S}_{j}}{S})$$

6 $$GainRatio\left(A\right)=\mathrm{Gain}(\mathrm{S},\mathrm{A})/{\mathrm{SplitInfo}}_{A}(\mathrm{S})$$

$Entropy\left(S\right)$ is dataset *S*. *v* is the different sub-data collections. $\frac{\left|{S}_{j}\right|}{\left|S\right|}$ is the ratio of the number of data in the *jth* sub-collection to the total data collections.

## Data Availability

The datasets generated from open access databases are available in the “Fishery Statistics Annual Report” repository, https://www.fa.gov.tw/cht/PublicationsFishYear/ and “National Food Consumption Database” repository, http://tnfcds.cmu.edu.tw/index.php?action=download&p=6. The datasets are analyzed by the proposed method of this study.

## Abbreviations

iAs: Inorganic arsenic
Cd: Cadmium
Co: Cobalt
Cr: Chromium
Cu: Copper
Fe: Iron
Mn: Manganese
Ni: Nickel
Pb: Lead
Sr: Strontium
Tl: Thallium
Zn: Zinc
V: Vanadium
HQ: Hazard quotient
USEPA: United States Environmental Protection Agency
EDI: Estimated daily intake
ADI: Acceptable daily intake
PTWI: Provisional tolerable weekly intake
BMDL: Benchmark dose lower confidence limit
MRL: Maximum residue limit
TDS: Total diet study
RfD: Reference dose
TFDA: Taiwan Food and Drug Administration
